# Treating Hypertriglyceridemia-Induced Pancreatitis With Intravenous Insulin and Plasmapheresis

**DOI:** 10.7759/cureus.30237

**Published:** 2022-10-12

**Authors:** Lay She Ng, Si Yuan Khor, Wern Lynn Ng

**Affiliations:** 1 Internal Medicine, Royal College of Surgeons in Ireland and University College Dublin School of Medicine, Malaysia Campus, George Town, MYS; 2 Internal Medicine, Michigan State University, Lansing, USA; 3 Internal Medicine, University of Pittsburgh Medical Center Harrisburg, Harrisburg, USA

**Keywords:** pancreatitis, plasmapheresis, insulin, case report, hypertriglyceridemia induced pancreatitis

## Abstract

Hypertriglyceridemic pancreatitis (HTGP) is well-known but it is extremely rare, especially in younger patients. The main treatment modalities for HTGP are apheresis and intravenous insulin. However, apheresis in severe HTGP is not well established and the efficacy of the treatment is lacking. Herein, we discuss a case of a 17-year-old female patient with no significant past medical history who initially presented to the emergency department with severe diabetic ketoacidosis (DKA) and was intubated due to severe metabolic acidosis and impending respiratory failure on arrival. Further investigation showed evidence of HTGP. Initially, her condition did not improve with intravenous insulin. However, a course of apheresis along with supportive care improved her condition drastically. Hence, this is a case report which showed the efficacy of concomitant use of insulin infusion and plasmapheresis in regard to treating HTGP. Outcomes of HTGP based on different treatment modalities are discussed in this literature as well. However, to date, there are no randomized studies to draw a solid treatment algorithm, thus further research on the most efficient treatment regimes is required for the management of HTGP.

## Introduction

Hypertriglyceridemia is an uncommon but well-known cause of acute pancreatitis (AP) [1]. Early clinical recognition of hypertriglyceridemic pancreatitis (HTGP) and prompt management (e.g., intravenous insulin, plasmapheresis, heparin, etc.) are crucial to reducing mortality and complications [1]. Plasmapheresis and intravenous insulin are the main therapy modalities that reduce serum triglyceride levels significantly [2-5]. However, plasmapheresis and insulin treatment in severe HTGP is not well established [2]. We report a case of a 17-year-old female with no significant past medical history that presented with severe HTGP and was successfully treated with intravenous insulin, hemodialysis, and plasmapheresis. 

## Case presentation

**Figure 1:**
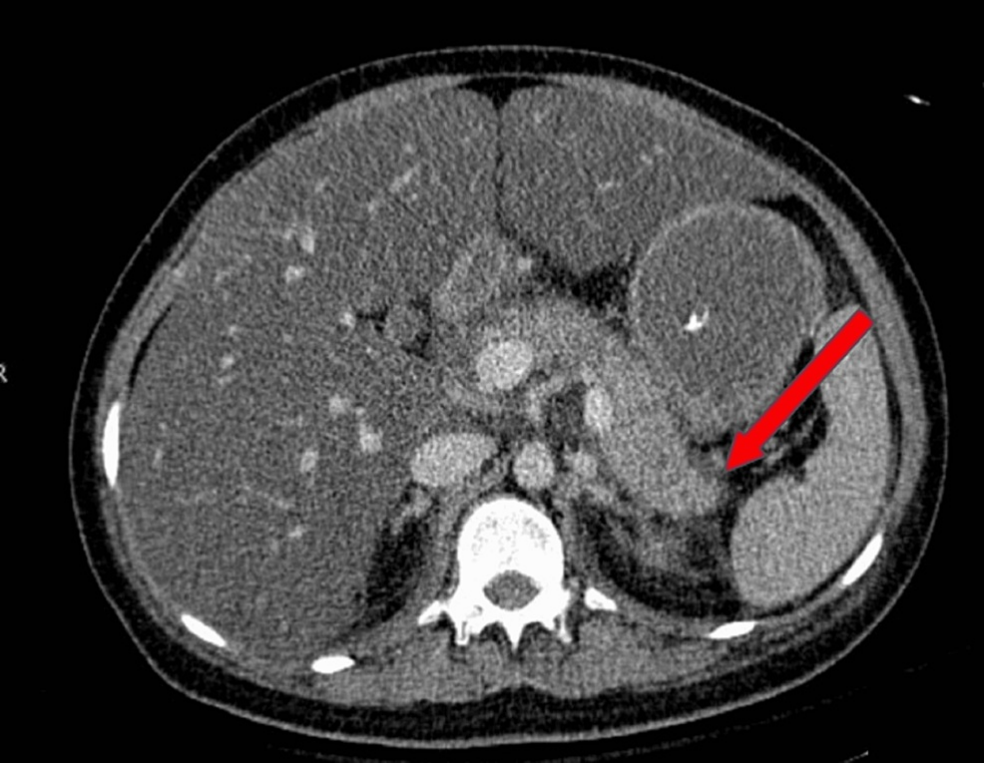
CECT abdomen which showed heterogenous enhanced hypodensity predominantly at the distal body and tail regions of the pancreas with peripancreatic fluid and streakiness consistent with AP (red arrow) CECT: contrast-enhanced computer tomography; AP: acute pancreatitis

A 17-year-old female with no significant past medical and family history presented with three days of generalized abdominal pain associated with the absence of bowel movements, polyuria, and polydipsia. On arrival at the emergency department, she had multiple episodes of emesis with brownish fluid vomitus. Initial vital signs revealed blood pressure 143/98 mmHg, heart rate of 120 bpm, respiratory rate of 30, a temperature of 37 °C and oxygen saturation of 99% on room air. Initial blood investigation was remarkable for white cell count of 27,000 cells/mL, capillary blood glucose - above readable value, capillary blood ketone 3 mmol/L, pH 7.1, pCO2 20 mmHg, serum bicarbonate 4.5 mEq/L, elevated amylase level 601 units/L (normal range: 40-140 units/L), and triglycerides levels were 21 mmol/L (1860mg/dL). Renal function, including creatinine and BUN levels, and liver function readings were within normal range. Contrast-enhanced computed tomography abdomen showed a heterogeneous enhanced hypodensity predominantly at the distal body and tail regions of the pancreas with peripancreatic fluid and streakiness consistent with AP (Figure 1). She was subsequently intubated in view of severe metabolic acidosis and impending respiratory collapse, and was admitted to the intensive care unit.

She was initially started on intravenous cefepime and metronidazole for gram-negative and anaerobes coverage respectively; intravenous insulin therapy and intravenous fluid for the treatment of DKA and AP. One session of hemodialysis was commenced in view of severe metabolic acidosis (bicarbonate levels of 10.6 mEq/L). After one session of hemodialysis, her blood results revealed that her triglyceride level was reduced from 21 to 9.13mmol/L (1860 to 808.7mg/dL), serum amylase level reduced from 569 to 279 units/L, and serum bicarbonate level increased from 10.6 to 16.5 mEq/L.

However, on Day 2 of admission, she developed systemic inflammatory response syndrome with a temperature of 38.8°C, tachycardia of 130 beats/minute, white blood cells count of 3,810 cells/mm3, and a score of two on the Modified Marshall Scoring System for Organ Dysfunction (her serum creatinine 211 increased from baseline 72 micromol/L) despite receiving antibiotics, fluid resuscitation, intravenous insulin therapy and one session of hemodialysis. Subsequently, she underwent a plasmapheresis session with 2.4 liters of fresh frozen plasma for severe lipemia and a second hemodialysis session. Her triglyceride level was reduced from 9.13 to 3.83 mmol/L (808.7 to 339.24mg/dL), her amylase level reduced from 279 to 110 units/L, and metabolic acidosis resolved (bicarbonate level increased from 15.4 to 21.2 mEq/L).

**Figure 2:**
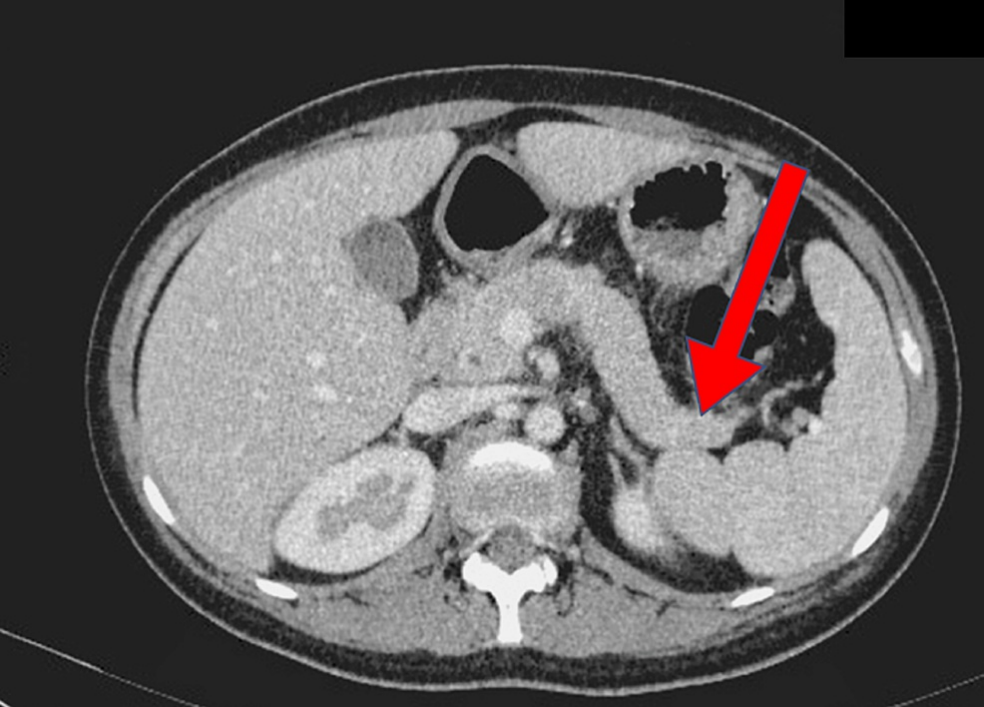
A repeated CT abdomen which showed resolution of pancreatitis (red arrow) CT: computer tomography

Her condition subsequently improved with intravenous insulin and gemfibrozil 300 mg two times a day. She was extubated on Day 5 and was transitioned to subcutaneous insulin on Day 7. She was clinically stable throughout and was discharged on Day 11 of her hospital stay with subcutaneous glargine 28 units at night, insulin aspart 10 units three times a day, fenofibrate 145mg daily, and lifestyle modification advice (weight loss as her initial BMI was 28 kg/m2, dietary modification (ie. die low in fat and carbohydrate) and aerobic exercise). She was followed up as an outpatient with a repeated contrast-enhanced computed tomography abdomen which showed resolution of pancreatitis (Figure 2) and her serum triglyceride level was reduced to 2.98 mmol/L after a month (Table 1). 

**Table 1:** Timeline summary of this case report TWC: total white cell; AP: acute pancreatitis; CECT: contrast-enhanced computer tomography

Time	Event	Finding/Treatment
3 days prior to admission	Symptoms onset	Generalized abdominal pain, absence of bowel movements, polyuria, and polydipsia.
Day 1 of admission	Intubation + hemodialysis	TWC 27,000 cells/mL; capillary blood glucose - above readable value; capillary blood ketone 3 mmol/L; serum bicarbonate 4.5 mEq/L; elevated amylase level 601 units/L; severe hypertriglyceridemia 21 mmol/L (1860mg/dL).
		CECT abdomen showed AP.
		After 1 session of hemodialysis : triglyceride level - 21 to 9.13mmol/L (1860 to 808.7mg/dL); amylase level - from 569 to 279 units/L; bicarbonate level - from 10.6 to 16.5 mEq/L.
Day 2 of admission	Plasmapheresis + hemodialysis	Triglyceride level was reduced from 9.13 to 3.83 mmol/L (808.7 to 339.24mg/dL); amylase level reduced from 279 to 110 units/L; metabolic acidosis resolved (bicarbonate level increased from 15.4 to 21.2 mEq/L).
		Started on intravenous insulin and gemfibrozil 300 mg two times a day
Day 5 of admission	Extubation	Continue intravenous insulin and gemfibrozil 300 mg two times a day
Day 11 of admission	Discharged	Subcutaneous glargine 28 units at night, insulin aspart 10 units three times a day, fenofibrate 145 mg daily, and lifestyle modification advice (weight loss, dietary modification, and aerobic exercise)
Day 30 since admission	Follow-up as an outpatient	CECT abdomen showed resolution of pancreatitis; serum triglyceride level was 2.98 mmol/L

## Discussion

Acute pancreatitis has a prevalence rate of 40-50 per 100,000 adults [1]. There is a 5% risk of developing acute pancreatitis with serum triglycerides >1000 mg/dL (11.3mmol/L) and up to 10-20% with triglycerides >2000 mg/dL (22.6mmol/L) [2]. Our patient developed acute pancreatitis with a serum triglyceride level of 1860mg/dL. Mortality associated with AP has decreased in the United States, with the most recent studies showing mortality of approximately 2%, but can be as high as 20-30% in patients with multiple organ failure [6]. Thus, early recognition and prompt management are needed to decrease the case fatality from AP. 

Primary (genetic) and secondary (acquired) disorders of lipid metabolism often coexist and cause hypertriglyceridemia, which may further induce pancreatitis. Primary hypertriglyceridemia often increases the risk of AP [7]. Primary hypertriglyceridemia includes type I (high chylomicrons), IV (high very low-density lipoprotein (VLDL)), and V (high chylomicrons and VLDL) dyslipidemias. Secondary (acquired) lipid metabolism disorder can be due to uncontrolled DM with DKA, obesity, alcoholism, hypothyroidism, medications such as hormonal supplementation with estrogen, selective estrogen receptor modulators (e.g., tamoxifen), pregnancy, second-generation antipsychotic medication, beta-blocker, etc [6].

In regards to the pathogenesis of HTGP, triglycerides themselves are not toxic, but they are hydrolyzed into lipotoxic free fatty acids by pancreatic lipases. The lipotoxic free fatty acids cause direct lipotoxicity and induce the inflammation of the pancreas via toll-like receptors causing pancreatitis and it may progress to systemic inflammation [8].

The clinical presentation of HTGP is similar to other forms of pancreatitis [6]. HTGP should be suspected in patients with risk factors of hypertriglyceridemia which include alcoholism, uncontrolled DM, familial hypertriglyceridemia, obesity, and pregnancy [6]. Physical findings vary depending on the severity of the disease. Patients may have fever, tachypnea, hypoxemia, hypotension, epigastric tenderness, abdominal distention, hypoactive bowel sound (due to inflammation causing an ileus), and icterus (due to edema of the head of the pancreas). Blood serum can become lactescent at high triglyceride levels [6].

**Figure 3:**
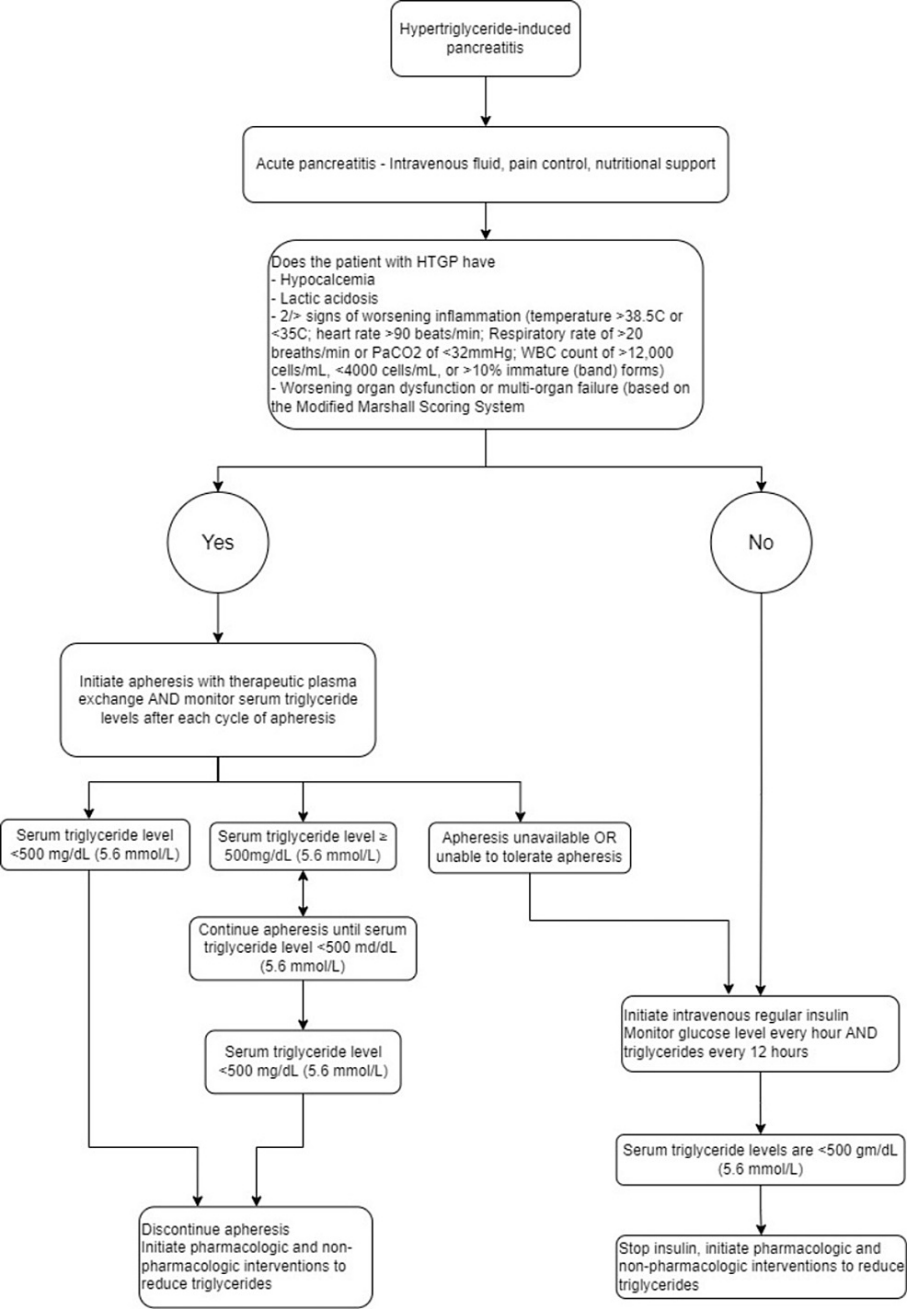
Suggested approach to the management of patients with HTGP This flowchart details a suggested approach to the management of patients with HTGP [3, 9, 12, 13]. HTGP: hypertriglyceridemic pancreatitis

Management for HTGP mainly includes the reduction of serum triglyceride levels and treatment of AP [2]. Insulin and apheresis are the main treatment modalities for HTGP. Different treatment modalities including fibrate, fish oil, and heparin have also been implemented to reduce serum triglycerides [9-11]. However, to date, randomized trials of the treatment efficacy are lacking. Other initial management of pancreatitis consists of supportive care with fluid resuscitation, pain control, and nutritional support which is similar to other causes of AP (Figure 3). Following the acute phase, lifestyle changes and drug therapy are important in the long-term management of HTGP to prevent recurrence.

The choice of initial therapy is based on the presence of worrisome clinical features and triglyceride levels. Worrisome clinical features include signs of hypocalcemia, lactic acidosis, two or more signs of worsening inflammation (temperature >38.5°C or <35.0°C; heart rate >90 beats/min; respiratory rate >20 breaths/min or PaCO2<32 mmHg; WBC count >12,000 cells/mL, <4000 cells/mL, or >10 percent immature (band) forms) or worsening organ dysfunction or multi-organ failure (based on Marshall scoring system for organ dysfunction) [13]. 

Patients with worrisome clinical features are treated with apheresis as initial therapy (Figure 3). Commencement of intravenous insulin therapy can take place if apheresis is unavailable or the patient is without worrisome features as mentioned. Our patient developed worrisome clinical features despite receiving antibiotics, fluid resuscitation, intravenous insulin therapy, and a session of hemodialysis, thus, a session of apheresis with hemodialysis was commenced. Apheresis rapidly removes the triglycerides, active enzymes, and pro-inflammatory cytokines including interleukin-1 and tumor necrosis factor-α; hence, it reduces the inflammatory process and contributes to faster recovery [14]. The mean removal rates for triglycerides after a single session of apheresis and two sessions of apheresis were 66.3% and 83.3% respectively [15]. Apheresis is highly recommended in AP patients displaying excessively elevated triglyceride levels. Our patient’s triglyceride level was reduced from 9.13 to 3.83mmol/L (808.7 to 339.24mg/dL), amylase level was reduced from 279 to 110units/L and metabolic acidosis resolved (bicarbonate level increased from 15.4 to 21.2mEq/L) after one session of apheresis and hemodialysis. Triglycerides should be monitored after each cycle of apheresis and apheresis should be continued until triglyceride levels are <500mg/dL. However, HTGP belongs to category-III indication (in which the optimum role of apheresis is not well established, and decision-making should be individualized) in American Society for Apheresis guidelines, due to its high cost and accessibility issues to apheresis [16].

In contrast, patients without worrisome clinical features are treated with intravenous insulin. The purpose of insulin is to promote intracellular triglyceride generation within adipocytes and to inhibit hormone-sensitive lipase in adipocytes, thus the level of fatty acids is decreased. Triglyceride levels in these patients should be monitored ever 12 hours and intravenous insulin should be continued until triglyceride levels are <500mg/dL. Glucose levels should be strictly monitored to prevent hypoglycemic episodes while receiving intravenous insulin treatment. It has been reported that insulin successfully treats patients with HTGP [3]. Thuzar et al. have described the approaches of different routes of insulin administration to patients with HTGP. Their study shows that mean serum triglyceride levels in patients with intravenous insulin alone were decreased by 40 ± 8.4% in the first 24 hours (from 94.3 ± 18.9 mmol/L to 57.6 ± 16.2 mmol/ L) and in patients with subcutaneous insulin by 23.5% (from 102 to 78 mmol/L) (p = 0.0003) [17]. In short, his study showed that intravenous insulin is more effective than subcutaneous insulin and insulin treatment is found to be efficient in HTGP management as well.

Once triglyceride levels are decreased to lower than 500mg/dL, patients require long-term management of hypertriglyceridemia to prevent the recurrence of HTGP with pharmacological and non-pharmacological management. Pharmacological management includes fibrates, omega-3 fatty acids, and niacin. Fibrates are the first-line medications for lowering triglycerides. Fibrates reduce 30-50% triglycerides with a concomitant increase in HDL [9]. Omega-3 fatty acids are dose-related and reduce 20-50% triglycerides. Niacin reduces 10-30% triglycerides. However, omega-3 fatty acids and niacin have not shown cardiovascular benefits [10]. Non-pharmacological management includes lifestyle modifications such as a low-fat diet, weight loss, and strict glycemic control. Our patient’s condition improved with intravenous/subcutaneous insulin and fibrates. Her CECT abdomen showed resolution of pancreatitis and her serum triglyceride level was reduced to 2.98 mmol/L after one month of treatment.

Heparin is one of the treatment modalities used in HTGP. It causes a transient nature of increment in circulating lipoprotein lipase levels, leading to a decrement in triglyceride levels [11]. Nonetheless, the efficacy of heparin in HTGP treatment is not known.

However, the proof of efficacy of the treatment is still lacking. More data on the efficacy is needed to conclude and draw a definitive treatment algorithm. A few case reports in the literature (Table 2) have demonstrated that both insulin therapy and plasmapheresis showed significant improvement in treating HTGP.

**Table 2:** Summary of literature regarding triglyceride levels and outcome of HTGP. TG: triglyceride

Case reports	Treatment modalities	Triglyceride level	Outcome
Aryal et al. [18]	Heparin + insulin intravenously	TG level 15 215 mg/dL	363 mg/dl on Day 6 of admission
Bajaj et al. [19]	Subcutaneous insulin + lipid-lowering drugs	TG level 12,234 mg/dL	TG level 1,824 mg/dL (day 4); TG level 465 (Day 8). Resolved pancreatitis but developed other complications due to prolonged hospitalization
Khalifa et al. [4]	1 session of hemodialysis + 8 sessions of plasmapheresis + full dose of hypolipidemics	TG level 1335 mg/dL	TG level (after one session): 934mg/dL TG (After 8 sessions of plasmapheresis): 1335 -> 545 mg/dL
Gayam et al. [20]	Insulin	TGs level 10 612 mg/dL	TG level (Day 2): 6120mg/dL; TG level (Day 7): 500mg/dL TG level (Day 8): Below 300mg/dL
Melnick et al. [5]	Intravenous insulin then plasmapheresis	TGs level >10,000 mg/dL	TG level (Day 5 with IV insulin): 6,069 mg/dL; TG level (one session of plasmapheresis) 2,055 mg/dL; (second session) 642 mg/dL
Current case study	Insulin + hemodialysis + plasmapheresis	TGs level 1893mg/dL	TG level (Day 3): 339mg/dL

## Conclusions

Early recognition and prompt management of HTGP are crucial to reducing mortality. The use of insulin and plasmapheresis are the active treatment modalities for HTGP that have been used along with symptomatic management for AP which include pain control, intravenous fluids, and bowel rest. Plasmapheresis rapidly removes plasma triglycerides, thus reducing the inflammatory process and contributing to the resolution of pancreatitis. Insulin plays an important role in reducing serum triglycerides. A few case reports in the literature have demonstrated the efficacy of insulin and apheresis use to treat HTGP. Our patient developed HTGP most likely due to uncontrolled DM with DKA causing severe hypertriglyceridemia; her condition improved after insulin infusion, hemodialysis, and plasmapheresis. In a nutshell, treating HTGP patients with insulin infusion and plasmapheresis concomitantly show the best result. However, further research and international consensus on the treatment are still needed. To date, there are no definite guidelines for HTGP treatment.
